# Carnitine Palmitoyltransferase II (CPT2) Deficiency in a Patient With Recurrent Rhabdomyolysis: A Case Report

**DOI:** 10.7759/cureus.76332

**Published:** 2024-12-24

**Authors:** Kevin A Lease, David Wang

**Affiliations:** 1 Internal Medicine, University of Missouri School of Medicine, Columbia, USA

**Keywords:** beta-oxidation, cpt2 deficiency, medium-chain triglycerides, non-traumatic rhabdomyolysis, rare genetic diseases

## Abstract

Carnitine palmitoyltransferase II (CPT2) deficiency is a rare genetic disorder that prevents the body from using long-chain fatty acids (LCFAs) for energy. We report a case of a 40-year-old male with a recent episode of rhabdomyolysis triggered by an illness. His liver function tests (LFTs) and creatine kinase (CK) levels were markedly elevated. His rhabdomyolysis improved in the hospital with supportive treatment. At follow-up appointments, it was found that he had labs consistent with CPT2 deficiency. Genetic testing confirmed a homozygous mutation in the CPT2 gene. This report highlights the importance of considering CPT2 deficiency as a cause of recurrent rhabdomyolysis, especially when triggered by non-traumatic causes.

## Introduction

Carnitine palmitoyltransferase II (CPT2) deficiency is a metabolic disorder that prevents the body from effectively using long-chain fatty acids (LCFAs) for energy [1]. CPT2 is a key enzyme facilitating the eventual beta-oxidation of LCFAs within the mitochondria. LCFAs are conjugated to coenzyme-A (CoA) by long-chain fatty acyl-CoA synthetase. The carnitine shuttle then transports these compounds across the outer and inner mitochondrial membranes (IMM). The carnitine shuttle consists of CPT2, carnitine acylcarnitine translocase, and carnitine palmitoyltransferase 1 (CPT1). CPT2’s main function is the transesterification of LCFAs. This allows their transport to the inner mitochondria for beta-oxidation [2]. Beta-oxidation is a catabolic process in which fatty acid chains are cleaved in the mitochondria to eventually produce ATP through the citric acid cycle. The process allows the body to utilize stored fats as an energy source and provides an alternative fuel source during periods of fasting, low glucose availability, or high energy demand [3]. It is one of the main ways the body maintains energy homeostasis [4].

There are three clinical variants of CPT2 deficiency: lethal neonatal, severe infantile, and myopathic [1]. The neonatal form presents with seizures, cardiac dysfunction, liver dysfunction, lethargy, and premature death. The severe infantile form presents a bit later and has similar manifestations as the neonatal form. In the myopathic form, patients typically present with episodes of muscle pain, stiffness, rhabdomyolysis, and myoglobinuria. These episodes are usually triggered by physical activity, fasting, or illness [5]. The cytotoxic effects seen in CPT2 deficiency are produced by increased cytoplasmic and mitochondrial ionized calcium as a result of direct damage to the plasma membrane or ATP depletion [6]. Individuals with the myopathic form of CPT2 deficiency inherit it in an autosomal recessive pattern [7]. We present a case of a patient who had recurrent episodes of rhabdomyolysis and was found to have CPT2 deficiency.

## Case presentation

A 40-year-old male with a history of rhabdomyolysis and exercise-induced asthma presented to the emergency department for a one-day history of dark urine and diffuse body aches. He had been at a family get-together two days prior where several family members had tested positive for Influenza. He had developed a cough around this time. His last episode of rhabdomyolysis had occurred around six years ago when he had been working out regularly. His brother also had an episode of rhabdomyolysis. He did not take any medications regularly. Cardiovascular and respiratory exams were normal. The urine appeared dark. He had no neurologic abnormalities. Vital signs were unremarkable. Labs upon admission were notable for mild hyponatremia (sodium: 131 mmol/L); potassium of 4 mmol/L; elevated aspartate aminotransferase (AST; 1024 U/L) and alanine aminotransferase (ALT; 231 U/L); normal alkaline phosphatase (ALP; 64 U/L); creatinine of 1.02 mg/dL; and elevated creatine kinase (CK; 58,317 U/L). There were no previous creatinine values in our medical records to review. The white blood cell (WBC) count was normal at 9.99 K/U/L. Hemoglobin was elevated at 17.8 g/dL, likely due to dehydration, and platelets were 205 K/U/L. Urinalysis (UA) showed a large amount of blood and only 0-2 red blood cells/high-powered field. Influenza A was positive. Fluorescent antinuclear antibody (FANA) was positive at 1:320 (Table 1).

**Table 1:** Lab values upon admission Notable values are highlighted in bold ALP: alkaline phosphatase; ALT: alanine aminotransferase; AST: aspartate aminotransferase; CK: creatine kinase; FANA: fluorescent antinuclear antibody; WBC: White blood cells

Variables	Value	Reference range
Sodium, nmol/L	131	136-145
Potassium, mmol/L	4	3.5-5.1
AST, U/L	1024	<40
ALT, U/L	231	10-50
ALP, U/L	64	40-129
Creatinine, mg/dL	1.02	0.7-1.2
WBC, K/uL	9.99K	3.5-10.5
Hemoglobin, g/dL	17.8	13.5-17.5
Platelets, K/uL	205K	150-450
CK, U/L	58317	20-200
FANA	1:320	<1:80

**Figure 1:**
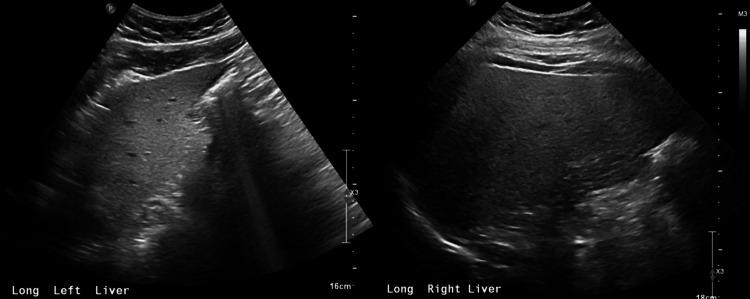
Abdominal ultrasound showing diffusely increased hepatic echogenicity No suspicious lesions were seen

Urine drug screen, alcohol, and acetaminophen levels were unremarkable. Hepatitis B and C tests returned negative. Intravenous fluids were started and CK levels were monitored. The patient’s urine color became less dark and his myalgias improved. His CK levels and liver function tests (LFTs) decreased throughout his hospitalization. Creatinine decreased to 0.77 on discharge. He was discharged on hospital day six and was seen in the clinic around a month later. Further labs and workup at that time showed normalization of his CK level and LFTs. Hemoglobin electrophoresis was unremarkable. Iron studies showed slightly low iron saturation. Reticulocyte count and hemoglobin were normal. The erythrocyte sedimentation rate (ESR) was slightly elevated. Abdominal ultrasound showed findings consistent with hepatic steatosis (Figure 1). Antinuclear antibody (ANA) testing was positive at 1:640, and the patient was referred to rheumatology. The rheumatologist suspected a metabolic myopathy and the patient underwent a metabolic myopathy panel, which showed elevated levels of hexadecanoylcarnitine (C16) and octadecanoylcarnitine (C18), a common finding in patients with CPT2 deficiency (Table 2) [4].

**Table 2:** Plasma concentration of carnitine and carnitine esters in the patient Elevated levels of multiple long-chain carnitine esters (C16 and C18) were found

Type of Carnitine	Concentration, nmol/ml	Reference range, nmol/ml
Acylcarnitine	6	5-30
Carnitine free	42	25-54
Carnitine total	48	34-78
Acetylcarnitine (C2)	4.66	2.00-17.83
Acrylylcarnitine (C3:1)	<0.02	<0.07
Propionylcarnitine (C3)	0.57	<0.88
Formiminoglutamate (FIGLU)	<0.01	<0.14
Iso-/butyrylcarnitine (C4)	0.16	<0.83
Tiglycarnitine (C5:1)	0.01	<0.11
Isovaleryl(2-methylbutyryl)carnitine (C5)	0.16	<0.51
3-OH-iso-/butyrylcarnitine (C4-OH)	0.02	<0.18
Hexenoylcarnitine (C6:1)	<0.01	<0.15
Hexanoylcarnitine (C6)	0.03	<0.17
3-OH-isovalerylcarnitine (C5-OH)	0.02	<0.1
Benzoylcarnitine	0.01	<0.1
Heptanoylcarnitine (C7)	0.01	<0.06
3-OH-hexanoylcarnitine (C6-OH)	0.01	<0.09
Phenylacetylcarnitine	<0.02	<0.29
Salicylcarnitine	<0.05	<0.09
Octenoylcarnitine (C8:1)	0.08	<0.88
Octanoylcarnitine (C8)	0.07	<0.78
Malonylcarnitine (C3-DC)	0.03	<0.26
Decadienoylcarnitine (C10:2)	<0.05	<0.26
Decenoylcarnitine (C10:1)	0.06	<0.47
Decanoylcarnitine (C10)	0.15	<0.88
Methylmalonyl-/succinylcarn (C4-DC)	0.03	<0.05
3-OH-decenoylcarnitine (C10:1-OH)	0.02	<0.13
Glutarylcarnitine (C5-DC)	0.03	<0.11
Dodecenoylcarnitine (C12:1)	0.03	<0.35
Dodecanoylcarnitine (C12)	0.09	<0.26
3-Methylglutarylcarnitine (C6-DC)	0.06	<0.43
3-OH-dodecenoylcarnitine (C12:1-OH)	0.02	<0.13
3-OH-dodecanoylcarnitine (C12-OH)	0.01	<0.08
Tetradecadienoylcarnitine (C14:2)	<0.02	<0.18
Tetradecenoylcarnitine (C14:1)	0.02	<0.24
Tetradecanoylcarnitine (C14)	0.08	<0.12
Octanedioylcarnitine (C8-DC)	0.02	<0.19
3-OH-tetradecenoylcarnitine (C14:1OH)	0.03	<0.13
3-OH-tetradecanoylcarnitine (C14-OH)	0.01	<0.08
Hexadecenoylcarnitine (C16:1)	0.02	<0.1
Hexadecanoylcarnitine (C16)	0.25 (elevated)	<0.23
3-OH-hexadecenoylcarnitine (C16:1-OH)	0.02	<0.06
3-OH-hexadecanoylcarnitine (C16-OH)	0.01	<0.06
Octadecadienoylcarnitine (C18:2)	0.07	<0.24
Octadecenoylcarnitine (C18:1)	0.17	<0.39
Octadecanoylcarnitine (C18)	0.14 (elevated)	<0.14
Dodecanedioylcarnitine (C12-DC)	0.02	<0.04
3-OH-octadecadienoylcarn (C18:2-OH)	<0.02	<0.056
3-OH-octadecenoylcarnitine (C18:1-OH)	0.01	<0.06
3-OH-octadecanoylcarnitine (C18-OH)	0	<0.03

The patient subsequently underwent genetics testing, which revealed a homozygous mutation in the CPT2 gene (CPT2 missense mutation at amino acid 113 in which serine is changed to leucine) leading to CPT2 deficiency. He was started on levocarnitine 1000 mg four times per day and medium chain triglycerides oil 15 milliliters twice per day. He was also counseled about dietary and behavioral interventions to try to avoid provoking rhabdomyolysis and has not had additional episodes to date.

**Figure 2:**
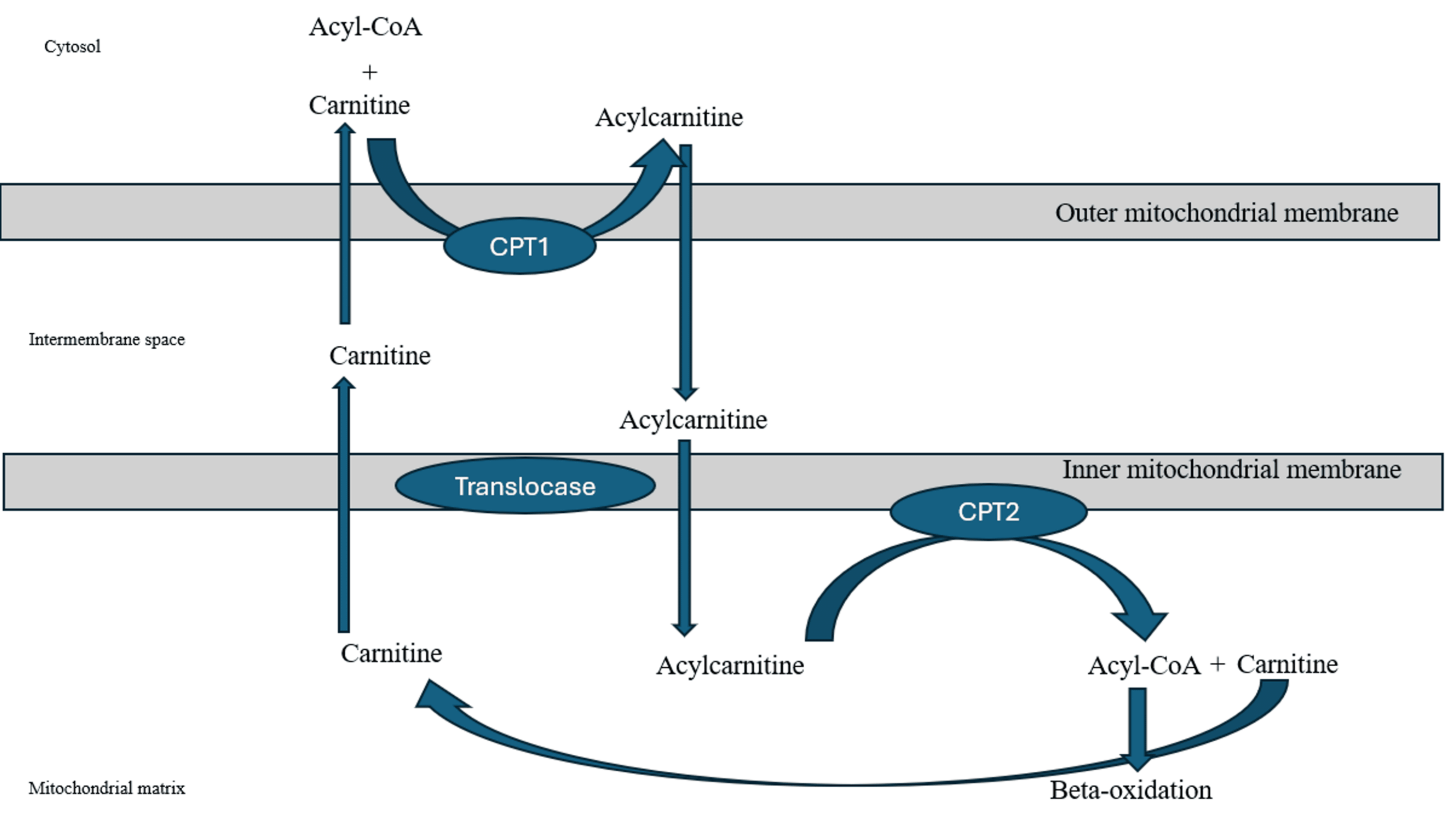
Diagram illustrating the transport of long-chain fatty acids in the mitochondria CPT2 helps convert long-chain acylcarnitines into Acyl-CoA. Acyl-CoA undergoes beta-oxidation to eventually generate energy Image credits: David Wang

Figure 2 presents a diagram illustrating the transport of LCFAs in the mitochondria.

## Discussion

CPT2 deficiency is the most common genetic disease of LCFAs even though though its exact incidence is unknown [8]. The myopathic form is the most common cause of hereditary myoglobinuria and 75% of those affected are male [9]. This deficiency results in acylcarnitine being unable to be fully transported through the IMM, leading to an inadequate generation of acyl-CoA and subsequent build-up of acylcarnitine [10]. The plasma concentration of acylcarnitine can be measured to aid in diagnosing CPT2 deficiency. Elevated levels of C16 and C18 acylcarnitines are characteristic of this disorder [11]. Replacement of serine with leucine at position 113 of the protein is found in a majority of patients with muscle CPT2 deficiency, but over 70 mutations of the CPT2 gene have been identified to potentially cause CPT2 deficiency [10].

An MRI study of individuals with LCFA disorders demonstrated elevated levels of short-TI inversion recovery (STIR) and T1-weighted (T1W) signal intensity, reflecting increased lipid accumulation and inflammation. STIR and T1W signal intensities were less prominent in the MRIs of CPT2 patients [12]. While our patient did not have an MRI during his workup, it would be interesting to see how it may be used as a diagnostic modality. Some of the most common triggers for attacks include exercise, infections, decreased nutritional intake, and a cold. There are also reports of attacks being triggered by certain medications including nonsteroidal anti-inflammatory drugs and high doses of diazepam and sodium valproate [5]. Our patient’s rhabdomyolysis was most likely triggered by his recent influenza infection.

There is no approved drug treatment for CPT2 deficiency although a low-fat diet enriched with carnitine and medium-chain triglycerides is recommended [8,13]. Increased dietary carnitine may help to decrease potentially toxic long-chain acyl-CoA. Metabolism of medium-chain triglycerides is independent of multiple enzymes including CPT2 [1]. Individuals with this condition should also be encouraged to avoid fasting, strenuous exercise, and extreme temperatures, all of which are associated with provoking rhabdomyolysis [14]. In cases of rhabdomyolysis, such as in our patient, aggressive hydration with intravenous fluids is recommended [15]. This report illustrates how detailed history, physical exam, and lab testing can help identify the cause of recurrent rhabdomyolysis. Clinicians should be mindful of CPT2 as a possible cause of rhabdomyolysis to facilitate appropriate treatment, especially in cases of rhabdomyolysis in the setting of fever, exercise, relatively mild illness, or fasting.

## Conclusions

We discussed a clinical case of myopathic CPT2 deficiency diagnosed in a middle-aged adult who presented with rhabdomyolysis following an influenza illness. The myopathic form is characterized by myalgia, myoglobinuria, and rhabdomyolysis. It can present later in life and is most commonly triggered by fasting, exercise, or illness. Genetic testing can confirm the diagnosis. The treatment largely involves avoiding conditions that tend to trigger episodes of rhabdomyolysis and dietary supplementation, which have thus far helped to prevent additional episodes in our patient. We hope this report will raise clinicians’ awareness of CPT2 deficiency as a possible cause of rhabdomyolysis as well as about some key diagnostic tests and treatments for the management of this condition.

## References

[1] Wieser T (1993). Carnitine palmitoyltransferase II deficiency. GeneReviews.

[2] Darras BT, Friedman NR (2000). Metabolic myopathies: a clinical approach; part I. Pediatr Neurol.

[3] Lee K, Kerner J, Hoppel CL (2011). Mitochondrial carnitine palmitoyltransferase 1a (CPT1a) is part of an outer membrane fatty acid transfer complex. J Biol Chem.

[4] Ivin N, Della Torre V, Sanders F, Youngman M (2020). Rhabdomyolysis caused by carnitine palmitoyltransferase 2 deficiency: a case report and systematic review of the literature. J Intensive Care Soc.

[5] Deschauer M, Wieser T, Zierz S (2005). Muscle carnitine palmitoyltransferase II deficiency: clinical and molecular genetic features and diagnostic aspects. Arch Neurol.

[6] Joshi PR, Zierz S (2020). Muscle carnitine palmitoyltransferase II (CPT II) deficiency: a conceptual approach. Molecules.

[7] Bonnefont JP, Djouadi F, Prip-Buus C, Gobin S, Munnich A, Bastin J (2004). Carnitine palmitoyltransferases 1 and 2: biochemical, molecular and medical aspects. Mol Aspects Med.

[8] Joshi PR, Deschauer M, Zierz S (2019). Phenotype of carnitine palmitoyltransferase II (CPT II) deficiency: a questionnaire-based survey. J Clin Neurosci.

[9] Castillo E, Medina D, Schoenmann N (2023). Myopathic carnitine palmitoyltransferase II (CPT II) deficiency: a rare cause of acute kidney injury and cardiomyopathy. Cureus.

[10] Joshi PR, Deschauer M, Zierz S (2014). Carnitine palmitoyltransferase II (CPT II) deficiency: genotype-phenotype analysis of 50 patients. J Neurol Sci.

[11] de Sain-van der Velden MG, Diekman EF, Jans JJ, van der Ham M, Prinsen BH, Visser G, Verhoeven-Duif NM (2013). Differences between acylcarnitine profiles in plasma and bloodspots. Mol Genet Metab.

[12] Diekman EF, van der Pol WL, Nievelstein RA, Houten SM, Wijburg FA, Visser G (2014). Muscle MRI in patients with long-chain fatty acid oxidation disorders. J Inherit Metab Dis.

[13] Lamhonwah AM, Olpin SE, Pollitt RJ (2002). Novel OCTN2 mutations: no genotype-phenotype correlations: early carnitine therapy prevents cardiomyopathy. Am J Med Genet.

[14] Longo N, Amat di San Filippo C, Pasquali M (2006). Disorders of carnitine transport and the carnitine cycle. Am J Med Genet C Semin Med Genet.

[15] Kodadek L, Carmichael Ii SP, Seshadri A (2022). Rhabdomyolysis: an American Association for the Surgery of Trauma Critical Care Committee Clinical Consensus Document. Trauma Surg Acute Care Open.

